# Evaluation of the effects of a diabetes educational program: a randomized clinical trial

**DOI:** 10.11606/S1518-8787.2018052007132

**Published:** 2018-01-29

**Authors:** Heloísa de Carvalho Torres, Ana Emília Pace, Fernanda Figueredo Chaves, Gustavo Velasquez-Melendez, Ilka Afonso Reis

**Affiliations:** I Universidade Federal de Minas Gerais. Escola de Enfermagem. Departamento de Enfermagem Aplicada. Belo Horizonte, MG, Brasil; II Universidade de São Paulo. Escola de Enfermagem de Ribeirão Preto. Departamento de Enfermagem Geral e Especializada. Ribeirão Preto, SP, Brasil; III Universidade Federal de Minas Gerais. Escola de Enfermagem. Programa de Pós-Graduação em Enfermagem. Belo Horizonte, MG, Brasil; IV Universidade Federal de Minas Gerais. Escola de Enfermagem. Departamento de Enfermagem Materno-Infantil e Saúde Pública. Belo Horizonte, MG, Brasil; V Universidade Federal de Minas Gerais. Instituto de Ciências Exatas. Departamento de Estatística. Belo Horizonte, MG, Brasil

**Keywords:** *Diabetes Mellitus*, Type 2, prevention & control, Self Care, Health Education, Health Programs and Plans, Evaluation of the Efficacy-Effectiveness of Interventions, Outcome and Process Assessment (Health Care), Diabetes Mellitus Tipo 2, prevenção & controle, Autocuidado, Educação em Saúde, Planos e Programas de Saúde, Avaliação de Eficácia-Efetividade de Intervenções, Avaliação de Processos e Resultados (Cuidados de Saúde)

## Abstract

**OBJECTIVE:**

Evaluate the effectiveness of a diabetes mellitus educational program in primary health care.

**METHODS:**

This cluster randomized trial was conducted in a sample of 470 people with type 2 diabetes mellitus from eight health units, randomly assigned to two groups: intervention (n = 231) and control (n = 239). The intervention group participated in the educational program composed of three strategies: group education, home visit, and telephone intervention. Simultaneously, the control group was monitored individually. Group monitoring took place over nine months in the year 2012. Clinical evaluations were performed at the initial time (T_0_), three (T_3_), six (T_6_) and nine (T_9_) months after the beginning of the intervention.

**RESULTS:**

After nine months of follow-up, 341 users remained in the study, 171 in the control group and 170 in the intervention group. The average age of users was 60.6 years. In both groups, statistically significant differences were observed in mean HbA1c levels over the follow-up time (p < 0.05). However, the mean HbA1c level at T_3_, T_6_ and T_9_ times were significantly lower among the people in the intervention group (p < 0.05).

**CONCLUSIONS:**

The educational program model developed was effective to improve the glycemic control of the intervention group participants.

## RESUMO

**OBJETIVO:**

Avaliar a efetividade do programa educativo em diabetes mellitus na atenção primária à saúde.

**MÉTODOS:**

Este ensaio clínico randomizado por conglomerados foi realizado em amostra de 470 pessoas com diabetes mellitus tipo 2, provenientes de oito unidades de saúde, alocadas aleatoriamente em dois grupos: intervenção (n = 231) e controle (n = 239). O grupo intervenção participou do programa educativo composto de três estratégias: educação em grupo, visita domiciliar e intervenção telefônica. Simultaneamente, o grupo controle foi acompanhado individualmente. O acompanhamento dos grupos ocorreu ao longo de nove meses no ano de 2012. Foram realizadas avaliações clínicas no tempo inicial (T_0_), três (T_3_), seis (T_6_) e nove (T_9_) meses depois do início da intervenção.

**RESULTADOS:**

Após nove meses de seguimento, permaneceram no estudo 341 usuários, 171 no grupo controle e 170 no grupo intervenção. A média de idade dos usuários era de 60,6 anos. Em ambos os grupos, observou-se diferenças estatisticamente significativas nos níveis médios de HbA1c ao longo do tempo de acompanhamento (p < 0,05). Porém, o nível médio de HbA1c nos tempos T_3_, T_6_ e T_9_ foi significativamente menor entre as pessoas do grupo intervenção (p < 0,05).

**CONCLUSÕES:**

O modelo do programa educativo desenvolvido foi efetivo para a melhora do controle glicêmico dos participantes do grupo intervenção.

## INTRODUCTION

Type 2 diabetes mellitus (DM) accounts for 90% to 95% of DM cases and can be attributed to the effects of population aging combined with an unhealthy lifestyle such as poor eating habits and sedentary lifestyle^1^. Brazil is the fourth country with the highest number of adults between 20 and 79 years of age with diabetes, and the first among South and Central America countries^14^ ^,^ ^15^. Diabetes mellitus care programs should involve behavioral, psychosocial, and clinical aspects, and consider the values, opinions, and experiences of people in developing knowledge and skills for physical activity practices, following a food plan in the sociocultural context of the person's life^1^ ^,^ ^3^ ^,^ ^4^ ^,^ ^20^ ^,^ ^24^ ^,^ ^26^ ^,^ ^30^.

The behavioral changes required to control the condition of diabetes, especially those related to non-pharmacological treatment, contribute to a low adherence to self-care, which is a challenge for both people with diabetes and professionals involved in health care^10^. Therefore, educational practices directed to self-care of the person with diabetes have been carried out in Brazil, mainly through lectures for awareness. However, these strategies have been shown to have a low impact on adherence to treatment and self-care^2^ ^,^ ^37^ ^,^ ^40^ ^,^ ^42^. Providing program models in health care is important to instrumentalize the person in decision making and responsibility for their health care in controlling the condition and preventing their chronic complications^10^ ^,^ ^38^.

Primary Health Care is a favorable scenario for the implementation of the diabetes educational program, which, in partnership with the University, has sought to develop pedagogical practices based on the user's approach, such as group education, home visit, and telephone intervention. Such measures create an environment conducive to dialogue and a process of reflection aimed at self-care, and therefore, a possible increase in the effectiveness of programs of this nature inserted at the primary health care level^13^ ^,^ ^16^ ^,^ ^27^ ^,^ ^39^.

The purpose of this study was to evaluate the effects of an educational program in diabetes glycemic control in the Primary Health Care of Belo Horizonte, the capital of the state of Minas Gerais, Brazil.

## METHODS

The study was a cluster-randomized clinical trial conducted in a sample of people with DM enrolled and clinically monitored at eight basic health units (BHU) in the city of Belo Horizonte, capital of the state of Minas Gerais, Brazil, and participants in the diabetes educational program started in the year 2012. The people were recruited through records in medical records of the Basic Health Units. In addition to having a diagnosis of type 2 DM, the inclusion criteria were age between 30 and 70 years and availability to attend the consultations performed throughout the study. The main exclusion criteria were illiteracy and chronic complications defined as renal failure, blindness, amputation of limbs, among others.

To calculate the unadjusted sample size (*m*) in each conglomerate, the Campbell et al.^2^ (2004) expression was used. To accommodate the cluster effect, the design effect was calculated as *DE* = 1 + (*n̅* - 1)ρ, where *n̅* is the mean cluster size and r is the intraclass correlation coefficient. In order to achieve the adjusted sample size (*n*) in each cluster, the value of *m* was multiplied by the design effect DE, whose effect is to increase the size of the sample to consider the possibility of lower variability among users within the same cluster than among users of different clusters. The values used in the calculation of the sample size were: significance level α = 0.05, test power ω = 0.90, standard effect size for glycohemoglobin *d* = 1, *n̅* = 100 and r = 0.008 (obtained from previous design data and similar to the data of this study). Thus, n = 47 users were calculated for each cluster (Basic Health Unit), which meant that each of the two study groups should have at least 188 participants at the end of the study. Considering a loss of up to 20% throughout the follow-up, each of the two study groups should have at least 235 participants at the start of the study.

Of the eight Basic Health Units, from which the study sample originated, four comprised the intervention group and four formed the control group. The allocation of each unit was done by lottery, using a computer program to generate random sequences with the numbers one to eight. The participants of the two groups were monitored through telephone contacts for nine months, following the program established by each unit and according to the type of strategy, which satisfied the individual needs and availabilities. The users allocated to the control group participated in the educational practices developed in the routine of the respective health units and maintained the conventional follow-up, performed in the Basic Health Units, through clinical care, according to the Primary Care protocol^23^. Finally, these users received two telephone calls, made by a nurse.

The program included the use of strategies: group education, home visit and telephone intervention, whose purpose was to insert users in the strategies that would give them better access to the teaching and learning process to strengthen self-care practices and goal setting. These strategies occurred concomitantly at three times (cycles), every three months. Most users who had a telephone (landline or cell phone) were accompanied by phone calls, in which the objective was to reinforce practices and minimize barriers to adherence to selfcare practices associated with following a healthy food plan and practicing physical activity.

Group education consisted of activities in small groups that discussed the topics: healthy eating, physical activity, feelings that may impair adherence to self-care practices, as well as the planning of goals inserted in the user's daily routine. Subjects were approached through playful and interactive dynamics, such as conversation maps “Understanding Diabetes”, “Healthy eating and physical activity” and “Glucose monitoring”^5^ ^,^ ^24^. Three meetings were held with an interval of one week. Each meeting lasted an average of two hours. In all the meetings, two professionals were present, usually a nurse and a nutritionist, who conducted the educational process in an interdisciplinary way, contributing to the interaction and reinforcement of contents, leading the user to benefit from a change of behavior and to become aware that their actions make a difference in treatment. Group education was comprised of nine face-to-face meetings held at the respective health units.

Participants who did not attend the meetings were contacted via telephone or had a home visit scheduled to perform the uncompleted activity.

The home visit discussed the needs faced by the user in the day to day regarding their condition from the understanding of the life context, in addition to addressing the emotional complications experienced by the user. The systematization of care, in turn, aimed to contemplate the needs of the user, promote their autonomy during the teaching-learning process for health care and lead to a comprehensive and humanized care. Each service had an average duration of 45 minutes and each user received two appointments, which were previously scheduled through telephone calls, according to the users’ interest, and performed by health professionals (nurses, physiotherapist, and nutritionist).

In the telephone intervention, the issues related to the food plan, physical activity, feelings, barriers and meeting of goals were addressed. The calls were made by a health professional and had an average duration of 25 minutes. We observed that the user needed to talk about their difficulties regarding self-care practices and took advantage of the telephone call to express their feelings of anguish when performing physical activity practices and following the healthy eating plan.

The primary endpoint was improvement in A1C glycated hemoglobin levels (HBA1c, in %), evaluated at baseline (T_0_), and at three-month intervals (T_3_, T_6_, and T_9_), and improved levels of total cholesterol (mg/dL), HDL cholesterol (mg/dL), LDL cholesterol (mg/dL), VLDL cholesterol (mg/dL) and triglycerides (), also monitored at the baseline and at T_3_, T_6_ and T_9_ times. To evaluate the HbA1c results, the parameter recommended by the Latin American Diabetes Association was used, according to which the values considered normal are in the range of 3.5 to 6.5%^13^.

The data were processed through the SPSS program (version 19.0), and double typed for data control and validation. Statistical analyses were carried out in the statistical programming environment R (version 3.0.1). To evaluate the effect of the intervention over time in each of the clinical variables, the Variance Analysis with repeated measures was used. To determine the need for more complex models of variance analysis, such as those that consider the effect of clusters, intra-class correlation coefficient values, considering the initial and final data of the clinical indicators, were evaluated and values smaller than 0.05 were considered low. The normality assumption of the data was verified by means of the Shapiro-Wilk test. In case of violation of the data variances homogeneity assumption in their original measurement scale, the Box-Cox transformation was used to correct the problem. Multiple comparisons were made by means of confidence intervals with the Bonferroni correction. To compare proportions, we used chi-square tests with Yates correction for independent samples and McNemar for dependent samples. To compare two means, the t-Student-Welch test was used, if the samples were independent, and the t-Student test was paired for dependent samples. In all analyses, a significance level of 5% was used.

This study was approved by the Research Ethics Committee of the Universidade Federal de Minas Gerais and the Municipal Secretary of Health of Belo Horizonte, state of Minas Gerais, through Report 0024.0.410.203-09.

## RESULTS

**Figure 1:**
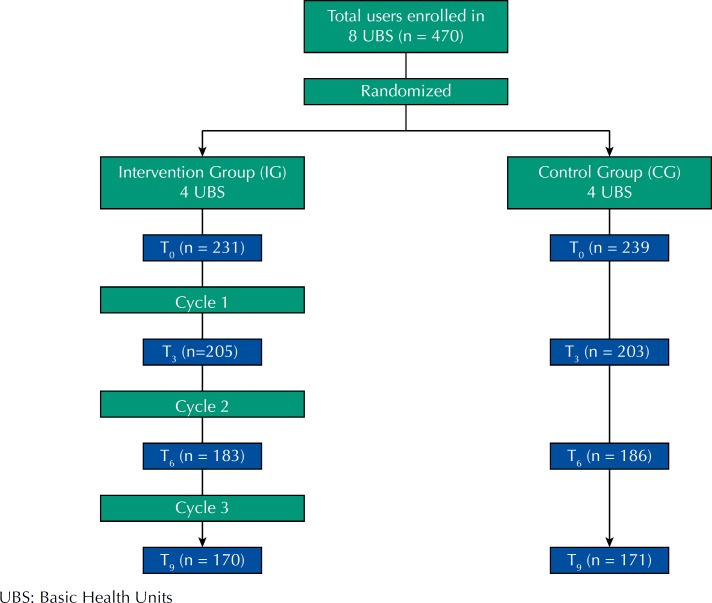
Study flowchart.

The number of participants who started the study was 470, from the eight basic health units, of which 239 (50.9%) were from the control group and 231 (49.1%) from the intervention group. The total loss after nine months of follow-up was 27.3%, remaining in the study 341 users, 171 in the control group and 170 in the intervention group (Figure 1). Among the reasons presented, most people did not participate due to lack of interest, change of address and others.

The control and intervention groups were considered comparable for clinical, anthropometric and sociodemographic variables, both at the baseline (T_0_) and at the end of the study (T_9_), except for the educational variable, for which the intervention group had a higher proportion of illiteracy than the control group (p < 0.020) (Table 1). Thus, losses can be considered random, except for schooling level.

**Table 1:** Mean (standard deviation) or counting (percentage) of sociodemographic and clinical variables of the users in the intervention and control groups at the baseline and at the end of the study (impact assessment of the losses).

Variable		Initial (T_0_)			Final (T_9_)		
		Control	Intervention	p^a^	Control	Intervention	p^a^
Gender		(n = 239)	(n = 231)		(n = 171)	(n = 170)	
	Female	161 (67.4%)	164 (71.0%)	0.452	118 (69.0%)	127 (74.7%)	0.294
	Male	78 (32.6%)	67 (29.0%)		53 (31.0%)	43 (25.3%)	
Occupation		(n = 230)	(n = 227)		(n = 167)	(n = 168)	
	Active	65 (28.3%)	55 (24.2%)	0.383	42 (25.1%)	39 (23.2%)	0.775
	Inactive	165 (71.7%)	172 (75.8%)		125 (74.9%)	129 (76.8%)	
Marital status		(n = 230)	(n = 226)		(n = 167)	(n = 167)	
	Had partner	127 (55.2%)	119 (52.7%)	0.649	93 (55.7%)	88 (52.7%)	0.660
	Without partner	103 (44.8%)	107 (47.3%)		74 (44.3%)	79 (47.3%)	
Education^b^		(n = 230)	(n = 225)		(n = 167)	(n = 167)	
	Illiterate	13 (5.7%)	38 (16.9%)	0.0003	13 (7.8%)	28 (16.8%)	0.020
	Literate	217 (94.3%)	187 (83.1%)		154 (92.2%)	139 (83.3%)	
Age (years)		(n = 230) 62.5 (10.5)	(n = 223) 62.1 (10.9)	0.682	(n = 167) 62.8 (10.5)	(n = 165) 63.4 (10.6)	0.475
Income (minimum wage)		(n = 230) 1.8 (1.9)	(n = 214) 1.9 (1.8)	0.846	(n = 167) 1.7 (1.9)	(n = 157) 1.7 (1.4)	0.827
Disease duration (years)		(n = 229) 9.8 (8.1)	(n = 221) 9.2 (8.1)	0.463	(n = 166) 9.9 (8.5)	(n = 163) 9.4 (8.3)	0.614
Body mass index (kg/m^2^)		(n = 230) 28.6 (5.1)	(n = 224) 29.2 (5.6)	0.272	(n = 167) 29.2 (5.3)	(n = 165) 28.6 (5.3)	0.389
Abdominal circumference (cm)		(n = 230) 101.5 (12.5)	(n = 222) 100.0 (13.7)	0.243	(n = 131) 101.5 (13.4)	(n = 143) 100.6 (12.9)	0.602

a T-Student-Welch for independent samples or chi-square test.

b The groups are statistically different in the percentage of illiterate people.

**Figure 2:**
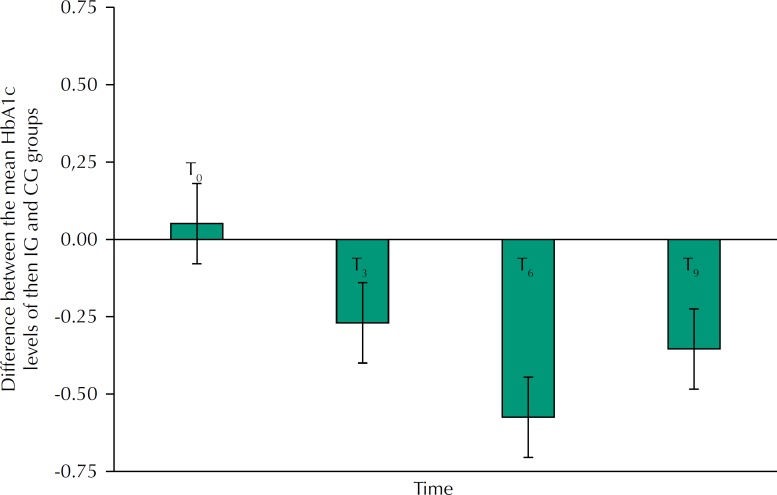
Average difference in HbA1c levels of the two groups over time: intervention group (IG) minus control group (CG). The intervals in the middle of the bars represent the 95% confidence intervals.

Regarding the clinical variables, the highest value obtained for the intra-class correlation coefficient was 0.038 (HDL variable at the baseline). Thus, there was no need to consider the cluster effect in data analysis, making it simpler. Table 2 presents the means and respective standard deviations of HbA1c levels and other clinical variables in the control and intervention groups during the study, as well as the results of the variance analysis. Figure 2 shows the difference in mean HbA1c levels between the intervention group and the control group at the four study times and their respective 95% confidence intervals. The two groups were considered comparable at the baseline for the clinical variables (p > 0.05; Figure 2). Over the follow-up time, there were statistically significant differences in mean HbA1c values in both the control and intervention groups. In the control group, mean hemoglobin A1c increased from initial time to T_3_ time and remained stable until the T_9_ time (p < 0.05; Table 2). In the intervention group, the mean hemoglobin A1c also increased between the initial time and T_3_, but at the T_6_ time, it returned to the mean value of the initial time and remained stable until the final time (T_9_) (p < 0.05). The mean value of HbA1c in the intervention group was considered statistically lower than the mean HbA1c value in the control group at T_3_, T_6_ and at the end of the study (T_9_) (p < 0.05) (Figure 2).

**Table 2:** Mean (standard deviation) of the biochemical variables along the follow-up of two study groups. P values refer to the F test of the variance analysis.

Variable	Group	Time			
		T_0_	T_3_	T_6_	T_9_
	Intervention	7.88^a^ (2.19) (n = 155)	8.31 (2.01) (n = 155)	7.95^a^ (1.86) (n = 155)	7.93^a^ (2.06) (n = 155)
HbA1c (%)		p < 0.05			
	Control	7.83^a^ (2.08) (n = 155)	8.59^b^ (2.06) (n = 155)	8.53^b^ (2.11) (n = 155)	8.29^b^ (2.22) (n = 155)
		p < 0.05			
	Intervention	188.48^a^ (55.41) (n = 155)	181.50^a^.^b^ (42.21) (n = 155	178.89^b^ (38.95) (n = 155)	180.07^a^.^b^ (41.39) (n = 155)
Total cholesterol (mg/dL)		p < 0.05			
	Control	188.13^a^ (45.11) (n = 155)	188.28^a^ (46.13) (n = 155	184.77^a^ (43.87) (n = 155)	183.38^a^ (39.84) (n = 155)
		p>0.05			
	Intervention	40.54 (31.87) (n = 155)	41.87^b^ (12.51) (n = 155)	41.69^b^ (10.48) (n = 155)	43.16^b^ (10.52) (n = 155)
HDL-C^d^ (mg/dL)		p < 0.05			
	Control	43.11^a^ (36.59) (n = 155)	59.95^b^ (35.50) (n = 155)	42.36^c^ (12.09) (n = 155)	42.97^c^ (10.86) (n = 155)
		p < 0.05			

a.b.c Equal letters on the same line indicate that the mean difference between two time periods is statistically zero (Student t-test paired with Bonferroni correction).

d Analysis was done using the logarithmic scale (Box-Cox transformation).

Regarding the mean values of triglycerides, total cholesterol, and their fractions, the two groups were also considered comparable at the baseline (p > 0.30). There was statistically significant variation in the mean values of total cholesterol in the intervention group over the follow-up time (p < 0.05; Table 2). However, the mean total cholesterol level in T_9_ was not considered statistically different from the initial mean level. In the control group, mean levels of total cholesterol over time were not considered statistically different (p > 0.05).

The mean HDL cholesterol level of the intervention group increased statistically between T_0_ and T_3_ and remained stable until T_9_ (p < 0.05). In the control group, there were no statistically significant differences in the mean level of HDL cholesterol between T_0_ and T_9_ (p > 0.05).

For LDL cholesterol, there was a statistically significant increase in mean levels in both groups (p < 0.05). In both groups, there was a statistically significant decrease in the mean VLDL cholesterol level between T_0_ and T_9_ (p < 0.05). At the mean triglyceride rates, no statistically significant differences were observed between follow-up times in any of the comparison groups (p > 0.05).

As for the percentage of users with altered hemoglobin A1c, the control and intervention groups were considered equal in the initial time (68.2% and 65.8%, respectively, p = 0.649) as well as in the final time (76.0% and 74.1%, respectively, p = 0.778). In both groups, the percentage of people with altered hemoglobin A1c can be considered statistically larger in the final time compared to the initial time (p values equal to 0.042 and 0.004 for the control and intervention groups, respectively).

## DISCUSSION

Education for self-care and the development of knowledge is a process with several challenges, especially in the presence of DM, a condition that affects people of all ages, with different levels of schooling and social and environmental bases^6^ ^,^ ^17^ ^,^ ^21^ ^,^ ^25^ ^,^ ^26^ ^,^ ^29^ ^,^ ^41^. One study^9^ aimed to investigate whether a person with DM would be able to take care of and make the necessary decisions for better control of the condition. People who studied full-time were more likely to understand the outcome of the exam and the effectiveness of early treatment in preventing long-term problems.

It is necessary, therefore, to use a constructivist approach of continuous learning, able to contribute to awakening the reflexive, critical and creative potential of both the professional and the person. This approach may be an option in the context of care for the person with the disease^6^ ^,^ ^34^ ^,^ ^36^.

Education based on dialogue favors the process of teaching-learning in the task of caring for oneself^4^. In this aspect, the educational practice following the process of co-responsibility allows the person to become aware of their care, and responsible for their actions. The process leads the person to reflect on their practice of care, allowing them to make their own choices^8^ ^,^ ^18^ ^,^ ^22^ ^,^ ^23^ ^,^ ^37^.

The results of this study suggest that the effects of the educational strategies used contributed to the maintenance of glycemic control throughout the study, as well as to its reduction when compared to the results of the control group. The effects of the intervention on reducing glycated hemoglobin levels (HbA1c) observed in the present study and during follow-up may have been underestimated, since users who had low educational level were mainly in the intervention group. People with DM who were part of the control group and who received conventional guidelines regarding medical consultation routines and information on healthy diet and physical activity showed increased levels of glycated hemoglobin from the sixth month of follow-up.

A second aspect of the results of this study is that, in the intervention group, there was a decrease in the values of the metabolic control variables. Because it was an educational intervention with three strategies (group education, home visit, and telephone intervention), it allowed greater flexibility for its conduction and may have favored this reduction, as found in other studies^9^ ^,^ ^31^ ^,^ ^32^ ^,^ ^34^ ^,^ ^35^. The idea of combining several educational strategies allows for greater convenience and the option of user involvement, alternative contact out of hours, outside working hours and prevents the user from going to the Health Center. Thus, associating different educational tools can reinforce the dialogue and bring cognitive gains to the issues that have been addressed.

In general, we observed that the expansion of educational and behavioral practices had the goal of involving and mobilizing the patient with diabetes, developing skills and strengthening educational activities for self-care required by the user. The patient proposed goals that contributed to help control the condition of diabetes, and most of them were able to comply with them in whole or in part. Effective communication through dialogue demonstrated the fulfillment of goals by the patient, and may be a way for Health professionals to act in the practices oriented towards the autonomy of health care and favoring co-responsibility.

In a systematic review, which included eleven clinical trials, the beneficial effects of intensive interventions directed at regular physical activities were shown in the results of HbA1c, lipids and blood pressure. The study emphasizes that intensive weight loss, regular physical activity and frequent contact with health professionals are required to achieve weight loss^6^ ^,^ ^7^ ^,^ ^14^ ^,^ ^17^.

In all the statistical analyses of the present study, the values of the probability of significance (p) were far from the reference value (0.05), which, together with the low values of the intraclass correlation coefficient, supports the option for the more statistical models since, in this case, considering the cluster effect would not change the conclusions obtained.

It should be noted that the duration of contact time between health professionals and users was 14 hours in the intervention group and 4 hours in the control group. A study^19^ points out that the time spent in the educational program is associated with obtaining new knowledge and improving the users’ self-care^11^ ^,^ ^12^ ^,^ ^28^ ^,^ ^33^ ^,^ ^39^.

Educational interventions with flexible strategies are presented as a viable alternative to raise awareness about diabetes care and to contribute to the maintenance or decrease of glycated hemoglobin levels and other indicators of metabolic function.
